# The Obesity and Fatty Liver Are Reduced by Plant-Derived *Pediococcus pentosaceus* LP28 in High Fat Diet-Induced Obese Mice

**DOI:** 10.1371/journal.pone.0030696

**Published:** 2012-02-17

**Authors:** Xingrong Zhao, Fumiko Higashikawa, Masafumi Noda, Yusuke Kawamura, Yasuyuki Matoba, Takanori Kumagai, Masanori Sugiyama

**Affiliations:** Department of Molecular Microbiology and Biotechnology, Graduate School of Biomedical Sciences, Hiroshima University, Hiroshima, Japan; University of Cordoba, Spain

## Abstract

We evaluated the effect of an oral administration of a plant-derived lactic acid bacterium, *Pediococcus pentosaceus* LP28 (LP28), on metabolic syndrome by using high fat diet-induced obese mice. The obese mice were divided into 2 groups and fed either a high fat or regular diet for 8 weeks. Each group was further divided into 3 groups, which took LP28, another plant-derived *Lactobacillus plantarum* SN13T (SN13T) or no lactic acid bacteria (LAB). The lean control mice were fed a regular diet without inducing obesity prior to the experiment. LP28 reduced body weight gain and liver lipid contents (triglyceride and cholesterol), in mice fed a high fat diet for 8 weeks (40%, 54%, and 70% less than those of the control group without LAB, and *P* = 0.018, *P*<0.001, and *P* = 0.021, respectively), whereas SN13T and the heat treated LP28 at 121°C for 15 min were ineffective. Abdominal visceral fat in the high fat diet mice fed with LP28 was also lower than that without LAB by 44%, although it was not significant but borderline (*P* = 0.076). The sizes of the adipocytes and the lipid droplets in the livers were obviously decreased. A real-time PCR analyses showed that lipid metabolism-related genes, such as *CD36* (*P* = 0.013), *SCD1* encoding stearoyl-CoA desaturase 1 (not significant but borderline, *P* = 0.066), and *PPARγ* encoding peroxisome proliferator-activated receptor gamma (*P* = 0.039), were down-regulated by taking LP28 continuously, when compared with those of the control group. In conclusion, LP28 may be a useful LAB strain for the prevention and reduction of the metabolic syndrome.

## Introduction

Metabolic syndrome is a cluster of abnormalities such as obesity, hypertension, dyslipidemia, and/or diabetes, which increases the risk of developing cardiovascular disease. The prevalence of this syndrome has been increasing in conjunction with industrial development, particularly in advanced nations. Of these risk factors, obesity, especially visceral adipose tissue accumulation, is considered as the major cause of cardiovascular disease [1]. The combination of dietary restrictions and physical exercise is effective for combating obesity and other metabolic disorders; however, the task is not easily achieved by most obese individuals.

Lactic acid bacteria (LAB) used for various fermented foods have therapeutic effects on human health, and their consumption has resulted in improvements of diverse gastrointestinal disorders, positive immunomodulation, and prevention of cancer [2]–[4]. LAB are major representatives of probiotics, which have been defined by the World Health Organization (WHO) as live microorganisms that, when administered in adequate amounts, confer a health benefit on the host. LAB is the generic name given to non-spore-forming Gram-positive bacteria that produce a large amount of lactic acid by the fermentation of various sugars and carbohydrates and can be roughly classified into two groups according to the difference in their living environments. One is derived from animals, and the other, from plants. Animal-derived LAB are generally used for the production of yogurt and cheese, and plant-derived bacteria are used in fermented foods, such as pickles, soy sauce, and grass silage. Previous studies suggest that plant-derived LAB are superior to animal-derived ones with regard to acid tolerance, immune-stimulating activity, and/or intestinal regulation [5]–[7]. Although various probiotic properties of LAB have been found until now, sufficiently potent probiotics to combat obesity have not been found yet. Some potent probiotics to prevent obesity might exist, as indicated by several reports on the serum lipid-lowering properties of lactobacilli [8]–[10]. Our previous clinical study suggests that a plant-derived strain, *Lactobacillus plantarum* SN13T (SN13T), isolated in our laboratory, is beneficial for improving liver function, as shown by measurements of serum aspartate aminotransferase (AST), alanine aminotransferase (ALT), and gamma glutamyl transpeptidase (γ-GTP), in addition to remarkable improvement of constipation [7]. We speculate that SN13T may have improved liver function in the clinical study by somehow reducing hepatic lipid contents in the study subjects with non-alcoholic or alcoholic fatty liver. In the present study, we, therefore, focused on contributions of the plant-derived LAB consumption to obesity assuming that it would change the lipid metabolism. Several LAB strains, including newly isolated *Pediococcus pentosaceus* LP28 (LP28) from the longan fruit, *Lactobacillus plantarum*, *Enterococcus mundtii*, and *Lactobacillus delbrueckii* subsp. *bulgaricus*, were preliminarily screened to determine whether or not they influence body weight in obesity-induced mice. We then proceeded using LP28 and SN13T and investigated their effects on body weight gain, visceral fat accumulation, plasma lipids, hepatic lipid contents, and the expression of some lipid metabolism-related genes in a high fat diet (HFD)-induced mouse model.

## Methods

### Ethics statement

Animal experiments were conducted in accordance with the “Guidelines for the Care and Use of Laboratory Animals” established by Hiroshima University, and all experimental procedures involving animals were approved by the Committee of Research Facilities for Laboratory Animal Science of Hiroshima University (Permit Number: A07-65).

### Isolation and identification of LP28

LP28 was newly isolated from longan fruit (*Euphoria longana*) by the method as described previously [11], identified by determining the entire 16S rDNA sequence by using previously reported methods [12]–[14], and compared with that of typical LAB obtained from the DNA Data Bank of Japan (DDBJ) Website [15].

### Experimental diets and LAB supplementation

The compositions of HFD (#D12492, Research Diets, Inc., New Brunswick, NJ) and regular diet (RD), which is a product named as “MF” manufactured by Oriental Yeast Co., Ltd., Japan, were shown in **Table 1**. Each cell mass of LP28 and SN13T, cultured in de Man-Rogosa-Sharpe (MRS) broth (Merck KGaA, Darmstadt, Germany) was collected by centrifugation and washed twice with phosphate-buffered saline (pH 7.4). The washed cell mass was lyophilized to make its powder and kept at −80°C until use. The lyophilized LAB was mixed with either RD or HFD at final concentrations of 1.25×10^9^ cfu/g. Diets of all groups were exchanged to maintain freshness every 7 d.

**Table 1:** Composition of high fat diet (HFD) and regular diet (RD).

	HFD	RD
Protein (% of energy)	20.0	26.2
Carbohydrate (% of energy)	20.0	60.4
Fat (% of energy)	60.0	13.3
Energy (kJ/g)	21.9	15.1

### Experimental animals

Male C57BL/6Jcl (SPF) mice, 6 to 7 weeks of age, were purchased from CLEA Japan, Inc. Mice were divided into seven groups of five mice each and were housed in plastic cages for each group under 40–60% humidity, controlled temperature (20–26°C), and 12 h light/dark cycles. Mice had free access to each diet and water. Six of the seven groups were fed HFD for 6 weeks to induce obesity before the oral administration of LAB. The remaining one group was continued to feed a RD as a lean control (RD/RD) mice without the induction of obesity. At the start of 8 week experimental period, each group was assigned to a HFD or RD, with or without LAB (**Table 2**). LAB were administered to mice for 8 weeks, and the food intakes were monitored by weighing the remaining diet from each cage when changing it. Body weight change of each mouse was recorded weekly, and blood samples were obtained from the mouse tail vein every 2 weeks for plasma lipid measurement. Food efficiencies were calculated as [body weight gain in 8 weeks] per [food intake in 8 weeks] [16]. After the experimental period, mice were killed by diethyl ether anesthesia, and followed by the isolation of livers and total visceral adipose tissues, such as mesenteric, epididymal, and retroperitoneal adipose tissue. We added two groups of two mice each (aged 6 to 7 weeks) to the seven groups (RD/RD, HFD/HFD-C, HFD/HFD-LP28, HFD/HFD-SN13T, HFD/RD-C, HFD/RD-LP28, and HFD/RD-SN13T), to check whether obesity was induced by feeding HFD for 6 weeks, since the mice in seven groups could not be used to determine the amount of total visceral adipose tissue, liver weight, and liver lipid contents at this point due to extirpation of the liver and adipose tissues. The additional two groups were also housed and fed either RD or HFD for 6 weeks at the same conditions as either RD/RD or other six experimental groups.

**Table 2:** Experimental design.

	Groups	Diet type during the obesity induction period (6 weeks)	Diet type during the experimental period (8 weeks)	LAB supplementations during the experimental period (8 weeks)
Lean mice	RD/RD	RD	RD	-
Obese mice fed a HFD	HFD/HFD-C	HFD	HFD	-
	HFD/HFD-LP28	HFD	HFD	LP28
	HFD/HFD-SN13T	HFD	HFD	SN13T
Obese mice fed a RD	HFD/RD-C	HFD	RD	-
	HFD/RD-LP28	HFD	RD	LP28
	HFD/RD-SN13T	HFD	RD	SN13T

HFD, high fat diet; RD, regular diet.

RD/RD, lean control group; HFD/HFD-C, obese control group fed a HFD; HFD/HFD-LP28, obese group fed the LP28-supplemented HFD; HFD/HFD-SN13T, obese group fed the SN13T-supplemented HFD.

HFD/RD-C, obese control group fed a RD; HFD/RD-LP28, obese group fed the LP28-supplemented RD; HFD/RD-SN13T, obese group fed the SN13T-supplemented RD.

### Plasma and liver lipid contents

The isolated livers were weighed after rinsed with phosphate-buffered saline and blotted dry with filter paper. Each liver was homogenized in 20 volumes of an extraction solution (chloroform: methanol = 2∶1; v/v) and agitated for 60 min at room temperature. The triglyceride and cholesterol levels in plasma and liver extraction were measured using the Triglyceride E-test Wako and Cholesterol E-test Wako (Wako Pure Chemical Industries, Osaka, Japan) according to the manufacturer's instructions.

### Histological analysis

Liver and visceral adipose tissue sections were 10% formalin-fixed, paraffin-embedded, and stained with hematoxylin-eosin (HE). Adipocyte numbers were counted in 4 different spots per mouse.

### Gene expressions

Quantitative real-time PCR analyses were performed for lipid metabolism-related gene expression in the liver. Briefly, total RNA was isolated from each liver using the SV Total RNA Isolation System (Promega, Madison, WI). The purity and integrity of RNA preparations were checked spectroscopically and by agarose gel electrophoresis. The total RNA was converted to cDNA using the RevertAid H Minus First-Strand TaKaRa RNA PCR Kit (Takara Bio Inc.) according to the manufacturer's instructions. Quantitative real-time PCR was performed with the iQ SYBR Green Supermix (TOYOBO Co. Osaka, Japan) and DNA Engine Opticon (Bio-Rad Laboratories, Hercules, CA). Primer sets for real-time PCR for *glyceraldehyde-3-phosphate dehydrogenase* (*GAPDH*), *CD36*, *Stearoyl-CoA desaturase-1* (*SCD1*), and *peroxisome proliferator-activated receptor gamma* (*PPARγ*) were obtained from Takara (*GAPDH*: MA050371, *CD36*: MA000930, *SCD1*: MA027073, *PPARγ*: MA029809).

### Statistical analysis

Statistical analyses were performed using SPSS (version 17.0: Statistical Package for the Social Sciences, SPSS Japan, Inc.). Due to non-normal distributions, liver weight, liver triglyceride, liver cholesterol, *CD36*, *SCD1*, and *PPARγ* were logarithmic transformed before the analysis. One-way ANOVA with Tukey's post hoc test was applied for all variables except for visceral fat, liver weight, liver triglyceride, and *CD36* in each diet group. The four variables were examined by Welch's ANOVA with the Tamhane post hoc test within each diet group, due to unequal variances. Dunnett's tests were done to compare each treatment group with RD/RD. The two-way ANOVA was also applied to evaluate the effects of diet, treatment, and interaction between the diet and treatment. To check the obese state at the starting point of experimental period, all mice available were used for the analyses; in short, 34 mice (three HFD/HFD groups, three HFD/RD groups, and additional two mice) fed HFD and 7 mice (RD/RD mice and additional two mice) fed RD were used for the analyses of body weight, plasma cholesterol, and triglyceride levels; and each of two additional mice fed HFD or RD were used for the amount of adipose tissue, liver cholesterol, and liver triglyceride contents. All data are presented as mean values with their standard errors. *P*<0.05 were considered significant.

## Results

### Obese state at the starting point of experimental period

Body weight after the 6 weeks obesity induction period was higher in the obese mice (HFD/HFD-C, HFD/HFD-LP28, HFD/HFD-SN13T, HFD/RD-C, HFD/RD-LP28, and HFD/RD-SN13T; 32.0±0.7 g) than that in RD/RD (27.1±0.6 g; *P* = 0.001, student *t* test). The plasma cholesterol level in the obese mice (1257±44 mg/L) were significantly higher than that in RD/RD (804±51 mg/L, *P*<0.001, student *t* test), whereas the plasma triglyceride levels did not alter in obese mice (1873±64 mg/L in obese mice and 1880±61 mg/L in RD/RD). The amount of abdominal adipose tissue, liver cholesterol, and liver triglyceride contents were 2.86±0.60 g, 6.6±2.2 mg/liver, and 27.9±2.9 mg/liver in mice fed HFD or 0.57±0.01 g, 5.1±0.8 mg/liver, and 23.3±5.1 mg/liver in mice fed RD, respectively, after the obesity induction period for 6 weeks.

### Anti-obesity effect of LP28

**Figure 1:**
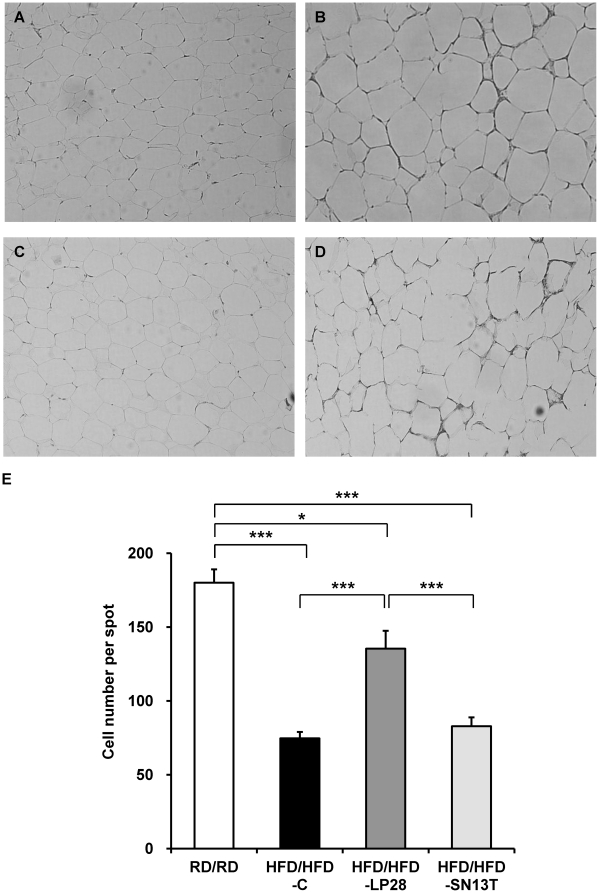
Effect of LAB supplementations on adipocyte size in obese mice. A, RD/RD; B, HFD/HFD-C; C, HFD/HFD-LP28; D, HFD/HFD-SN13T. Magnification, ×400. E, mean values of cell number per spot with their SE for 4 distinct areas per mouse. *P<0.05, ***P<0.005.

The body weight gain of HFD/HFD-LP28 was reduced by 40.7% during 8 weeks when compared to HFD/HFD-C (*P* = 0.018), whereas that of HFD/HFD-SN13T did not differ from HFD/HFD-C (Table 3). The reduction of weight gain in HFD/HFD-LP28 was started at week 2 (data not shown. Student *t* test, *P* = 0.032 versus HFD/HFD-C at the time point). The HFD/RD groups (HFD/RD-C, HFD/RD-LP28, and HFD/RD-SN13T) in which obesity had been induced by a HFD, followed by changing to a RD at the starting point of LAB administration, quickly returned to a normal body weight with reduced plasma cholesterol level (Table 3). Contrary to the result from the HFD/HFD groups, the body weight gain of HFD/RD-LP28 was not significantly lower than HFD/RD-C or HFD/RD-SN13T. There were no significant differences in the amounts of food intake and energy intake among the same diet groups, which had been recorded to determine whether or not their appetites had been depressed by supplement of LAB (Table 3). Food efficiency was significantly lower in HFD/HFD-LP28 than HFD/HFD-C and HFD/HFD-SN13T (*P* = 0.023 and *P* = 0.011, respectively). Plasma cholesterol levels in both the HFD/HFD and HFD/RD groups were not influenced by the consumption of LP28 and SN13T. The plasma triglyceride levels were unchanged by diet or LAB (Table 3). Two-way ANOVA showed a significant effect to visceral fat amounts in the diet and interaction between the diet and treatment. According to the analysis within the same diet group, the visceral fat in HFD/HFD-LP28 tended to be reduced when compared with that in HFD/HFD-C (*P* = 0.076). In the HFD/HFD-C group, HFD for additional 8 weeks induced 10.8 g of body weight gain and approximately 1.2 g of visceral fat gain, when compared with the starting point of experimental period (visceral fat amount: 2.86 g). However, although the body weight gain was 6.4 g by the LP28 treatment, the visceral fat was constant. In contrast, no quantitative difference of visceral fat among HFD/RD groups was observed. Judging from these observations, LP28-administrative effect may be different depending on the nutritional environment. The HFD/RD groups (HFD/RD-C, HFD/RD-LP28, and HFD/RD-SN13T) lost most of the visceral fat accumulation, which was formed during 6 week period of a high fat load prior to the experimental period (Table 3). Judging from the changes in adipocyte number per spot, the adipocyte size, which had become enlarged in HFD/HFD-C, was restored in HFD/HFD-LP28 but not in HFD/HFD-SN13T (**Figure 1**).

**Table 3:** Body weight gain, food efficiency, serum lipids levels, serum adiponectin level, visceral fat accumulation, liver weight, and liver lipid contents after the oral administration of LAB for 8 weeks^1^.

	Lean mice	Obese mice fed a HFD during the experimental period			Obese mice fed a RD during the experimental period			Two-way ANOVA (P)		
Variables	RD/RD	HFD/HFD-C	HFD/HFD-LP28	HFD/HFD-SN13T	HFD/RD-C	HFD/RD-LP28	HFD/RD-SN13T	Diet	Treatment	Interaction
Initial body weight (g)^2^	27.9±0.4	32.8±2.1	31.2±1.2	31.7±1.3	30.4±2.6	33.5±1.4	31.7±1.2	-	-	-
Final body weight (g)	32.4±0.5	43.5±3.0***	37.6±2.2	42.8±1.7***	31.5±1.7	33.7±1.1	31.7±0.7	<0.001	NS^3^	0.021
Body weight gain (g)	4.4±0.1	10.8±1.0***	6.4±0.9†	11.0±0.9***	1.1±1.0	0.2±0.8**	0.0±0.8**	<0.001	0.006	NS
Food intake (g/mouse per week)^4^	24.2±0.3	18.0±0.4***	17.7±0.5***	17.6±0.4***	21.9±1.1	25.8±1.7	25.4±1.9	<0.001	NS	NS
Energy intake (kJ/mouse per week)	364±5	394±9	389±11	386±10	330±17	388±26	382±29	NS	NS	NS
Food efficiency (g gain/g intake)	0.023±0.001	0.075±0.007***	0.045±0.007* †	0.078±0.006***	0.006±0.006	0.001±0.004*	0.000±0.004*	<0.001	0.004	NS
Serum total cholesterol (mg/L)	742±72	1494±120***	1448±147***	1645±114***	761±83	803±41	996±116	<0.001	NS	NS
Serum triglyceride (mg/L)	1803±50	2305±141	2165±144	2058±246	1982±176	1797±67	2329±253	NS	NS	NS
Serum adiponectin (mg/L)	24.6±0.6	34.6±2.5	35.1±4.5	24.6±2.9	25.4±3.8	20.9±2.6	21.1±1.7	0.001	NS	NS
Visceral fat (g)	0.95±0.09	4.03±0.48***	2.67±0.46***	3.53±0.17***	0.82±0.06	1.18±0.26	1.15±0.14	<0.001	NS	0.019
Liver weight (g)	1.12±0.05	1.40±0.08	1.28±0.05	1.54±0.17*	1.33±0.05	1.27±0.06	1.32±0.07	NS	NS	NS
Liver triglyceride (mg/liver)	37.3±3.9	136.2±13.3***	63.0±5.5** †††	189.5±26.1***	26.5±4.5	31.5±2.2	40.1±3.7	<0.001	<0.001	<0.001
Liver cholesterol(mg/liver)	4.8±1.0	8.9±1.3	2.7±0.7†	10.2±2.1*	4.5±0.5	4.8±0.7	6.6±1.2	NS	0.013	NS

1 Data are mean ± SE, n = 5.

2 Initial body weight was at the starting point of LAB supplementations.

3 Not significant.

4 The weights of supplemented LAB were excluded from the values of food intake.

*P<0.05.

**P<0.01,

***P<0.005 *vs.* RD/RD.

† *P*<0.05,

††† *P*<0.005 *vs.* HFD/HFD-C and HFD/HFD-SN13T.

### Reduction of fatty liver by LP28

**Figure 2:**
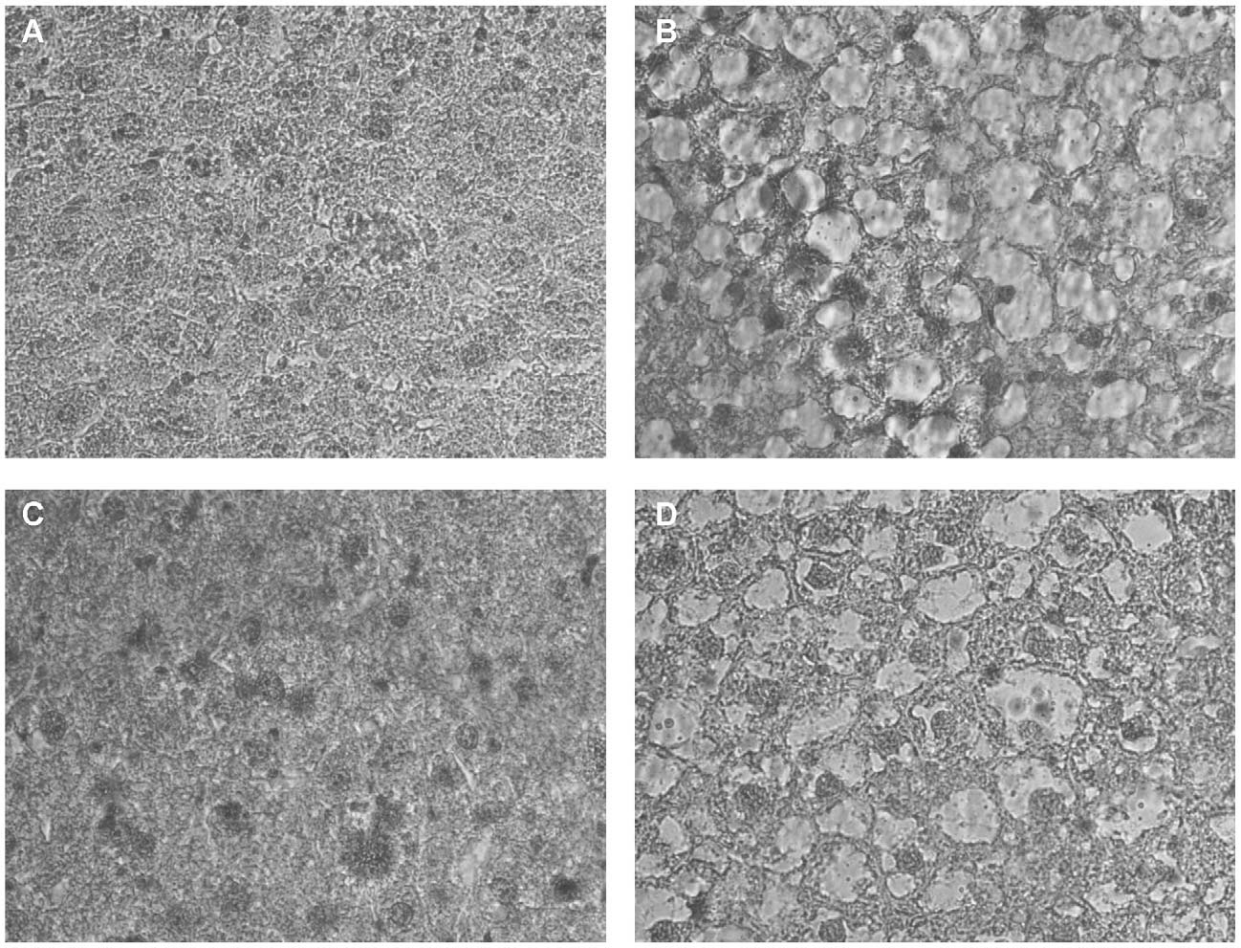
Effect of LAB supplementations on hepatic lipid contents in obese mice. A, RD/RD; B, HFD/HFD-C; C, HFD/HFD-LP28; D, HFD/HFD-SN13T. Magnification, ×400.

Triglyceride contents in the livers from HFD/HFD-C and HFD/HFD-SN13T showed a 3.7- (*P*<0.001) and 5.1-fold (*P*<0.001) higher than that from RD/RD, respectively, being in a condition referred to as “fatty liver” (Table 3). Although liver triglyceride content in HFD/HFD-LP28 was still higher than that of RD/RD (1.7-fold, *P* = 0.007), it was significantly lower when compared with HFD/HFD-C and HFD/HFD-SN13T (*P*<0.001 and *P*<0.001, respectively). Mice switched from the HFD to the RD for 8 weeks had liver triglyceride levels which were not different from the RD/RD. Hepatic cholesterol content in HFD/HFD-LP28 was also significantly less than those in HFD/HFD-C and HFD/HFD-SN13T (*P* = 0.021 and *P* = 0.012, respectively; Table 3). Moreover, the micrograph of the liver tissue sections shows that many massive lipid droplets were present in the HFD/HFD-C hepatic tissues, and had obviously shrunk in the HFD/HFD-LP28 liver, whereas SN13T did not affect the size of the lipid droplets in hepatic cells (**Figure 2**), in accordance with the results of the liver triglyceride content.

### Gene expression signature in hepatic cells in response to LP28

The real-time PCR analysis for liver tissues isolated from HFD/HFD-C and HFD/HFD-LP28 groups showed that the *CD36*, *SCD1*, and *PPARγ* expressions in HFD/HFD-LP28 were down-regulated when compared with those in HFD/HFD-C, as shown in **Table 4**. The *CD36* expression was highly induced in HFD/HFD-C in contrast to the RD/RD group (*P*<0.001); however, the induced expression of *CD36* in HFD/HFD-C was significantly suppressed in HFD/HFD-LP28 (*P* = 0.013) but not in HFD/HFD-SN13T. *SCD1* expression in HFD/HFD-LP28 was lower than that of HFD/HFD-C (*P* = 0.066), and *PPARγ* expression was reduced in HFD/HFD-LP28 when compared with HFD/HFD-C and HFD/HFD-SN13T (*P* = 0.039 and *P* = 0.044, respectively). Although two-way ANOVA showed significant treatment effects and no interaction effect between diet and treatment on *CD36* and *PPARγ* expressions, no significant reductions in the *CD36* and *PPARγ* expressions were observed in HFD/RD-LP28 group when compared with HFD/RD-C group.

**Table 4:** *CD36*, *SCD1*, and *PPARγ* mRNA expression levels in the liver after the oral administration of LAB for 8 weeks, which was examined by using the real-time PCR^1^^,^ 2.

	Lean mice	Obese mice fed a HFD during the experimental period			Obese mice fed a RD during the experimental period			Two-way ANOVA (P)		
Variables	RD/RD	HFD/HFD-C	HFD/HFD-LP28	HFD/HFD-SN13T	HFD/RD-C	HFD/RD-LP28	HFD/RD-SN13T	Diet	Treatment	Interaction
*CD36*	0.7±0.2	34.3±10.8***	5.5±1.9§	9.0±3.3***	3.8±1.3	1.6±0.4	4.4±1.5	<0.001	0.028	NS^3^
*SCD1*	2.9±0.8	6.4±2.2	2.2±0.4	4.6±2.0	9.2±2.2*	9.4±2.4*	6.0±1.8	0.002	NS	NS
*PPARγ*	2.8±0.8	4.6±1.1	1.5±0.3†	4.5±1.4	4.3±1.0	3.3±0.7	4.5±1.1	NS	0.019	NS

1 Data are mean ± SE, n = 5.

2 The data are expressed relative to *GAPDH* mRNA.

3 NS, Not significant.

*P<0.05.

***P<0.005 *vs.* RD/RD.

§ *P*<0.05 *vs.* HFD/HFD-C.

† *P*<0.05 *vs.* HFD/HFD-C and HFD/HFD-SN13T.

## Discussion

We have previously shown that SN13T had significant capacity to enhance immune responses and to improve gastrointestinal conditions and liver function [6], [7]. In this study, however, a newly isolated LAB, *Pediococcus pentosaceus* LP28, was demonstrated to be a potent anti-obesity probiotic, in contrast to the SN13T. We observed that LP28 killed by autoclaving at 121°C for 15 min is ineffective to reduce the obesity (10.3±0.6 g and 8.9±0.4 g weight gains in 8 weeks in control and heat-killed LP28 groups, respectively. *P*>0.05).

To evaluate the anti-obesity effects of the LAB strains, we arranged three sets of conditions for the mice. In one, we used normal mice (RD/RD) fed a typical diet for the entire experimental period; in the second, obese mice were fed a HFD (HFD/HFD-C, HFD/HFD-LP28, and HFD/HFD-SN13T); and, in the third, mice were fed a HFD for 6 weeks of obesity induction period and followed by feeding RD for 8 weeks during experimental period (HFD/RD-C, HFD/RD-LP28, and HFD/RD-SN13T). The results indicate that 8 week intake of a proper nutrient diet was sufficient to restore the body weight as well as the visceral fat and hepatic lipid storage.

The *CD36* expression in liver was also recovered in the HFD/RD groups (no significant difference is observed from RD/RD), however, *SCD1* expression levels were even increased in HFD/RD-C and HFD/RD-LP28 (*P* = 0.029 and *P* = 0.015 *vs.* RD/RD by Dunnett's test, respectively). This indicates that the nutritionally balanced food easily and quickly reduces fat accumulation regardless of the expression levels of *SCD1*. LP28 intake remarkably suppressed body weight gain caused by an extraordinarily fatty diet. The extent of weight gain suppression was 14.1% of initial body weight within only 8 weeks with LP28 supplementation, which is more than the range of 5% to 10% of weight reduction recommended by the U.S. National Heart, Lung, and Blood Institute, the WHO, and the Canadian Obesity Guidelines in order to reduce health risk of chronic disease [17]–[19]. Moreover, LP28 did not abnormally reduce the body weight of the HFD/RD groups, indicating that LP28 is effective only when nutrients are in excess. In this study, each mouse was administered approximately 3×10^9^ cfu/day of LAB, which is equivalent to 4.5×10^11^ cfu/day in human, when calculated by using the body surface area normalization method [20]. Although the currently used dosages of probiotics are 10^8^∼10^11^ cfu/day, the optimum dosage required for probiotics have not reached to a consensus [21]. Clinical study should be required to determine the effective dosage of LP28 in human. Body weight gain reduction by LP28 was accompanied by diverse changes, which included decrements of visceral fat amount, adipocyte size, lipid droplet size in liver, hepatic triglycerides contents, hepatic cholesterol contents, and hepatic gene expressions. However, LP28 had no effect on blood lipids, triglycerides and cholesterol. In addition, we evaluated plasma adiponectin concentrations, but no significant change was observed. It is possible that plant-derived LAB administered orally might adsorb dietary cholesterol or other lipids, which might then be excreted with feces. However, the cholesterol and fatty acid contents in feces were not increased in the HFD/HFD-LP28 group (data not shown).

The ingredient in RD may not match that in HFD. In this study, we performed the Dunnett's test to compare each of three HFD/HFD and three HFD/RD groups to lean control mice (RD/RD). Although this analysis is the comparison between different diet types, an aim of these comparisons is to know that whether the results from these experimental groups are close to that from RD/RD. A leading conclusions, which the intake of LP28 reduces the body weight gain and liver lipid contents, are obtained from the comparisons among the same diet group, such as among HFD/HFD-C, HFD/HFD-LP28, and HFD/HFD-SN13T, by the Tukey's test or the Tamhane test. Therefore, even if the ingredients in HFD are different from that in RD, it is clear that the intake of LP28 has these anti-obesity effects.

To elucidate the mechanism that combats obesity by LP28, we screened altered expressions of genes participating in lipid metabolism and biosynthesis. The expression level of CD36, which functions as a transporter for fatty acid and oxidized low-density lipoproteins [22], [23], is very low in normal liver tissue. *CD36* was drastically enhanced in a HFD-induced fatty liver and nonalcoholic fatty liver in humans, and these elevated levels of *CD36* expression correlated with hepatic fat contents [24], [25]. Conversely, forced expression of hepatic CD36 without a HFD caused hepatic steatosis [24]. It has also been reported that the development of fatty liver was prevented in *CD36* null mice [26]. The result as shown in Table 4 is agreeable with those from previous reports. Hepatic *CD36* expression in obese mice was considerably enhanced and paralleled by increased hepatic fat content. Upon LP28 administration, *CD36* expression and fat storage in the liver are concurrently reduced. These observations suggest that CD36 is associated with the prevention of hepatic steatosis achieved by LP28 intake.

A research group showed that SCD1 is predominantly expressed in liver and converts saturated fatty acids to monounsaturated fatty acids, which are major substrates for the synthesis of triglycerides, cholesterol esters, and other lipids [27]. *SCD1*-deficient mice have shown resistance to diet-induced obesity, increased insulin sensitivity, reduced hepatic lipid accumulation, and/or even increased energy expenditure [28]–[30]. Thus, *SCD1* may be a key gene to treat obesity. Our results suggest that the resistance to weight gain and hepatic fat accumulation as a consequence of LP28 consumption against a HFD load were caused, at least partially, by *SCD1* down-regulation in liver.

Furthermore, *PPARγ* expression was reduced in the liver of HFD/HFD-LP28 mice. It is known that PPARγ is highly expressed in adipose tissue, but it is also present in many other types of cells. It has been previously shown that PPARγ, which has wide effects on the lipid metabolism, plays a role in insulin sensitivity, fatty acid storage in adipocytes, and adipose differentiation [31]. However, liver PPARγ regulates triglyceride homeostasis and contributes to hepatic steatosis, accumulating triglycerides in the liver [32]. These results are consistent with our findings, which show reduced expression of hepatic *PPARγ* parallel with the prevention of fatty liver in the HFD/HFD-LP28 group. The combination of the regulation of these genes, *CD36*, *SCD1*, and *PPARγ*, in HFD/HFD-LP28 mice might comprehensively contribute to decreased fat accumulation and enhanced energy expenditure, followed by body weight loss.

In conclusion, LP28 was demonstrated as a potent anti-obesity probiotic. The precise mechanisms by which LP28 down-regulates the expressions of *CD36*, *SCD1*, and *PPARγ* remain to be accurately described and we are currently seeking to find the answers. However, our results offer evidence that plant-derived LAB strain, LP28 might be used as a supplement in foods that could effectively prevent obesity, hepatic steatosis, and metabolic syndrome.
